# Hydrogen extraction as a sustainable method for the recovery of phenolic compounds from tea wastes

**DOI:** 10.1007/s13197-025-06254-7

**Published:** 2025-02-24

**Authors:** Muhammed Allam Elnasankasim, Ayhan Çiğdem, Tunahan Engin, Duried Alwazeer

**Affiliations:** 1 Department of Food Engineering, Hatay Mustafa Kemal University, Hatay, 31060 Turkey; 2 Research Center for Redox Applications in Foods (RCRAF), Iğdır University, Iğdır, 76000 Turkey; 3 Innovative Food Technologies Development, Application, and Research Center, Iğdır University, Iğdır, 76000 Turkey; 4 Department of Bioengineering and Sciences, Postgraduate Education Institute, Iğdır University, Iğdır, 76000 Turkey; 5 YÖK 100/2000 Ph.D. Program, Iğdır, Turkey; 6 Department of Nutrition and Dietetics, Faculty of Health Sciences, Iğdır University, Iğdır, 76000 Turkey

**Keywords:** Tea waste, Hydrogen-rich water, Molecular hydrogen, Extraction, Sustainability, Phenolic recovery

## Abstract

The significant production of tea waste globally raises environmental concerns. Tea waste can be valorized by extracting its phytochemicals. In this study, the recovery of phenolic substances, flavonoids, and antioxidants from the black tea wastes using two types of hydrogen-rich water (HRW): hydrogen bubbling and magnesium-water reaction (Mg water), besides ethanol/water (50/50, % vol), ethanol/Mg water (50/50, % vol), and pure water (control) was investigated. The best extraction yield was obtained for HRW (30.13%). The best levels of phenolics (TPC), flavonoids (TFC), and antioxidants (DPPH and ABTS) were found for HRW extracts, followed by ethanol/Mg water (50/50). The levels of TPC, TFC, DPPH, and ABTS increased by 193.05, 210.56, 49.21, and 86.60%, and by 59.70, 33.46, 28.66, and 58.25% when HRW and Mg water was used as a solvent instead of pure water, respectively, in the extraction. The maximum levels of phenolic acids (p-coumaric acid, chlorogenic acid, gallic acid) and flavonoids (rutin and epicatechin) were found in HRW extracts. Hydrogen extraction can be proposed as a sustainable method to extract phenolic substances from agri-food waste.

## Highlights

- Huge production of tea generates significant quantities of phenolic-rich tea waste.
- The highest extraction yield was for HRW.
- The highest phenolics, flavonoids, and antioxidants were for HRW extracts.
- The levels of TPC, TFC, and antioxidants increased by about 2 folds by HRW.
- HRW is a green method for extracting phenolic substances from agri-food wastes.

## Introduction

A timely and sustainable solution seems necessary in the face of the global food security crisis, exacerbated by climate change and its ‎devastating effects on crop production and food availability. Additionally, due to the gradual increase in the world’s population and ever-growing consumption, a significant amount of agri-food waste, such as tea waste, is produced (Gunduzalp 2016).

Tea, a globally popular beverage, is a significant contributor to the food industry’s agri-food waste. ‎‎The 2021 global tea production reached a staggering 6.33 million tons (Nations 2022), resulting in 5 million tonnes substantial waste production (Kumar et al. 2023). The research findings can potentially revolutionize the tea ‎industry by offering a sustainable solution for utilizing tea waste.‎ Tea waste has been proposed as agricultural fertilizer and raw material to produce caffeine, catechin, and other phenolic substances (Ozarslan et al. 2022).

Tea leaves contain more than 4000 chemical components and are one of the plants containing vast amounts of flavonoids (Gübür 2015). The amount of polyphenols in tea leaves varies depending on the oxidation process applied during tea processing (Okinda Owuor et al. 2006).

Different extraction methods are used to enhance the recovery of phytochemicals, such as phenolic compounds. ‎Some methods need costly equipment, while others necessitate additional ‎preparation steps, increasing energy, solvent, cost, and time consumption.‎ Various extraction methods have been used to recover phytochemicals from tea and tea waste, such as classical (Mariya John et al. 2006), microwave-assisted (Li and Jiang 2010), ultrasonic-assisted (Horzic et al. 2012), high-pressure (Jun 2009), and supercritical fluid (Icen and Guru 2009) methods. However, using sustainable and cost-effective techniques is critical to preserving the planet’s natural resources.

Hydrogen (H_2_) is a non-toxic, inert, and reducing gas. H_2_ possesses more than 36 physical, chemical, and biological properties, of which the antioxidant property is the most important in food processing (Alwazeer 2024a). Due to its small size and high diffusibility, H_2_ can penetrate many membranes, tissues, and plastic materials. These specific properties of H_2_ opened the door for researchers to evaluate its potential applications in various fields, including health (Alwazeer 2024b; Xie and Song 2024), food and crops (Alwazeer and Engin 2022; Russell et al. 2024), agriculture (Zulfiqar et al. 2021; Alwazeer and Çiğdem 2022), ecology and environment (Köktürk et al. 2022a, b), and, of course, energy. Recently, the ability of H_2_ to enhance the phenolic, flavonoid, anthocyanin, and antioxidant extraction from different plant materials has been reported (Alwazeer and Elnasanelkasim 2023; Alwazeer et al. 2023a, b, c; d; Ceylan et al. 2023; Alwazeer 2023). Due to the massive generation of tea wastes worldwide, this study aimed to valorize them by evaluating the efficiency of hydrogen-rich water extraction as a sustainable method to recover phenolic substances from black tea wastes. The results can help develop a bio-based circular carbon economic model and a Bio-E3 concept, creating technology promoting the economy, environment, and employment (Hsiao and Hu 2024).

## Materials and methods

### Chemicals

The chemicals (DPPH, ABTS, Trolox, ascorbic acid, quercetin, 3,4,5 trihydroxybenzoic acid, potassium persulfate, Folin–Ciocalteu reagent were purchased from Merck (Darmstadt, Germany) and Sigma (MO, USA).

### Black tea waste preparation

The black tea waste was prepared in the laboratory, as shown below. A dry black tea was obtained from a local market. The black tea infusion was prepared by boiling 25 g of dry black tea leaves in one liter of water for 5 min. The tea leaves (wastes) were then separated and dried at 35 °C. The dried tea wastes were milled into powder and stored at -80 °C.

### Phenolic extraction

The extraction procedure was performed according to the method described by Alwazeer and co-workers (Alwazeer et al. 2023d). Briefly, 1 g tea waste powder was mixed with 20 mL of solvent [(pure water prepared by Millipore Milli-Q^®^ Direct 8 Water Purification System, USA), hydrogen-rich water (HRW), magnesium water (Mg water), ethanol/Mg water (50/50, % vol), and ethanol/pure water (50/50, % vol)], followed by a 24-hr incubation at 35 °C. The evaporation of solvents was then performed.

### Determination of total phenolic content

The total phenolic content (TPC) in tea waste extracts was determined according to the method described by Alwazeer and co-workers (Alwazeer et al. 2023d).

### Determination of total flavonoid content

The total flavonoid content (TFC) in tea waste samples was measured according to the method described by Alwazeer and co-workers (Alwazeer et al. 2023d).

### DPPH scavenging activity

The DPPH scavenging activity of tea waste samples was measured according to Alwazeer and co-workers (Alwazeer et al. 2023d).

### ABTS scavenging activity

The ABTS scavenging activity‎ of tea waste samples was measured according to the method described by Alwazeer and co-workers (Alwazeer et al. 2023d).

### HPLC analysis

The profile of phenolic substances was determined by the HPLC method described by Alwazeer and co-workers (Engin et al. 2025). An HPLC-DAD instrument (1260 Infinity, Agilent Technologies, USA) and a reverse phase C18 column (ACE Generix, particle size 250 × 4.6 mm, 5 μm particle size) were used in phenolic profile analysis. A volume of 20 µL of the filtered tea extract solution (0.45 μm membrane filter) was injected at a flow rate of 0.8 mL/min. A gradient elution system with a mobile phase composed of two solvents, A (1% vol, orthophosphric acid in water) and B (acetonitrile), at a total run time of 40 min, was applied. The gradient program started with 83% A and then changd to 60% and 83% at 30 mn and 35 min, respectively. Phenolic compounds were detected at 350/200 nm wavelength.

### Statistical analysis

Data were analyzed by one-way ANOVA using GraphPad Prism 9 Software. Turkey’s post hoc tests were followed by Duncan’s multiple comparison test using the IBM SPSS Statistics 26 package program. The significant differences were statistically considered at a level lower than 0.05. The whole experiment was duplicated, and the measurements were triplicated (*n* = 3).

## Results and discussion

### Extract yield

The extraction yield varied according to the type of solvent. The maximum extraction yield was obtained for HRW tea waste extracts at 30.13%, followed by ethanol/Mg water (50/50) at 21.86%, whereas the minimum yield was noticed for pure water at 16.23% (*P* < 0.05) (Table 1). Similar results were reported for olive leaves, where the maximum extraction yield was shown for HRW by an increase rate of 34.42%, followed by ethanol/Mg water (50/50) with an increase rate of 29.33%, whereas the minimum was shown for pure water by an increase rate of 19.27% (Alwazeer et al. 2023d).

**Table 1:** The extraction yield of the black tea waste using different solvents

Solvent	Extraction yield	
	Extract (mg)	Extract (%)
Pure water	162.3^e^	16.23
Mg water	207.4^c^	20.74
Hydrogen-rich water	301.3^a^	30.13
Ethanol/Mg water (50/50)	218.6^b^	21.86
Ethanol/water (50/50)	185.8^d^	18.58

^a−d^ different lowercase letters mean a significant difference between samples at *P* < 0.05

Hydrogen gas can easily pass through the plant cell’s cytoplasm membrane due to its high diffusion rate, reaching all compartments and organelles of the plant cell and increasing the separation of phenolics from plant materials via different possible mechanisms (Alwazeer 2023). The extraction yield of tea waste obtained with Mg water, in which the amounts of Mg^2+^ were high, was lower than that of HRW extracts. Tea phenolic substances can be partially retained by combining with ions such as Ca^2+^ and Mg^2+^ (Danrong et al. 2009).

### Total phenolic content (TPC)

The TPC values of tea waste samples prepared using different solvents are shown in Table 2. HRW tea waste extracts exhibited the highest TPC values, followed by ethanol/Mg water (50/50) samples with 163.70 and 134.06 mg GAE/g, respectively (*P* < 0.05). Additionally, using aqueous ethanol as a solvent increased the extraction of phenolics compared with pure water.

**Table 2:** Effect of solvent type on the recovery of phenolics, flavonoids, and antioxidants of black tea waste extracts

Parameter	Pure water	Mg water	Hydrogen-rich water	Ethanol/water (50/50)	Ethanol/Mg water (50/50)
Total phenolic content (mg GAE/g extract)	55.86 ± 0.64^e^	89.21 ± 0.99^c^	163.70 ± 1.98^a^	75.50 ± 1.65^d^	134.06 ± 1.96^b^
Total flavonoid content (mg QE/g extract)	19.04 ± 0.44^e^	25.41 ± 1.03^c^	59.13 ± 0.76^a^	22.29 ± 0.24^d^	40.66 ± 0.52^b^
DPPH scavenging activity (mg AAE/g extract)	15.18 ± 0.30^e^	19.53 ± 0.04^c^	22.65 ± 0.06^a^	19.01 ± 0.14^d^	21.99 ± 0. 04^b^
ABTS scavenging activity (mg TE/g extract)	14.25 ± 0.34^e^	22.55 ± 0.14^c^	26.59 ± 0.19^a^	21.89 ± 0.02^d^	24.58 ± 0. 05^b^

^a−d^ different lowercase letters show a significant difference between samples at *P* < 0.05

**Fig. 1:**
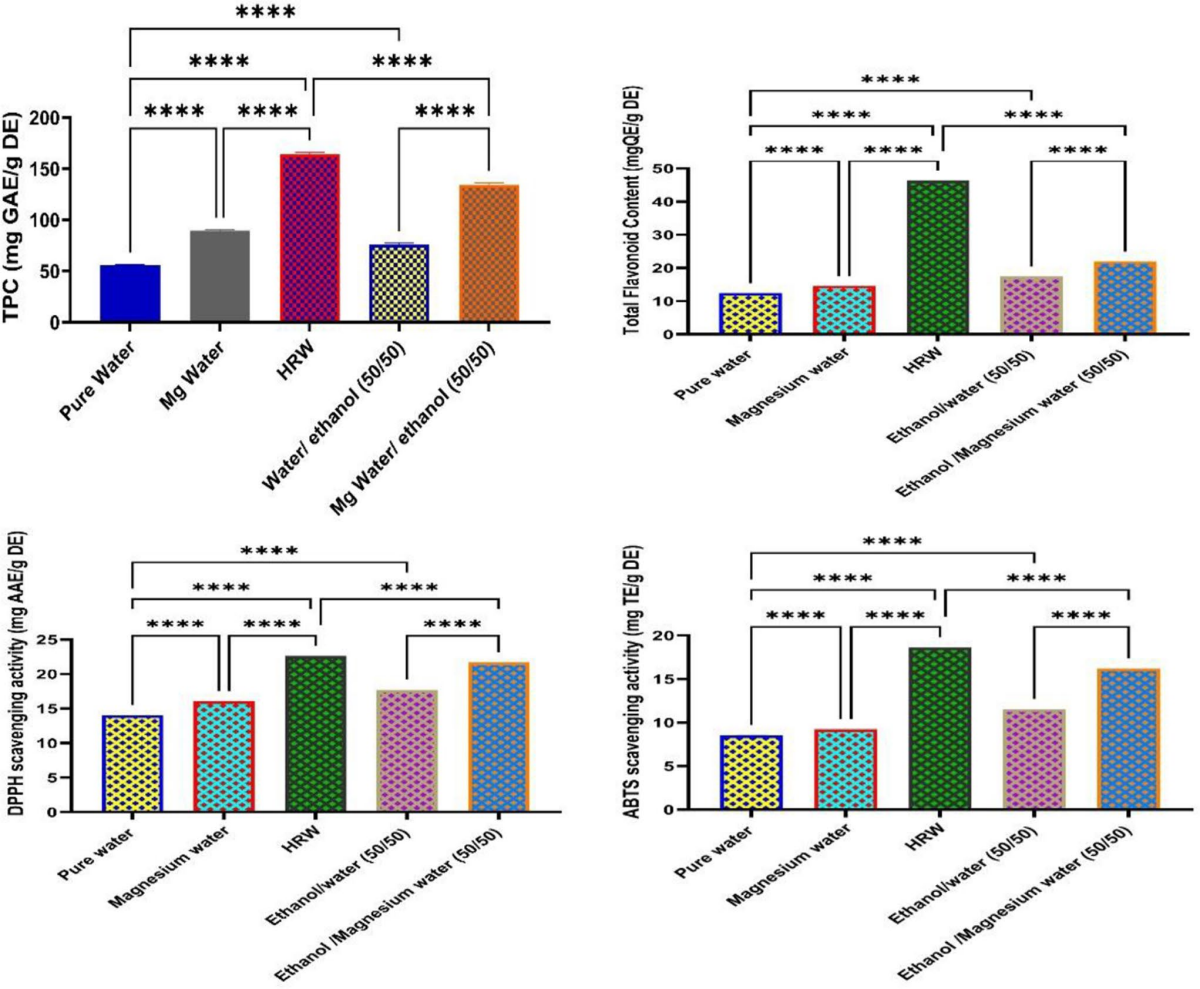
Phytochemicals of black tea waste extracts prepared by various solvents [(pure water), hydrogen-rich water (HRW), magnesium water (Mg water), ethanol/water (50/50, % vol), and ethanol/Mg water (50/50, % vol)] (****P < 0.0001)

Results show that the infusion of H_2_ into water by bubbling method for preparing HRW provided better TPC levels than the Mg-water reaction method. The TPC values increased by 193.05% for HRW samples compared to the pure water ones. However, using Mg water instead of pure water to prepare aqueous ethanol effectively enhanced the phenolic extraction (Fig. 1; Table 2). This led to a rise in the TPC level by only 77.56% (*P* < 0.05) (Table 3). The results show that the use of HRW alone or with ethanol effectively improved the recovery of phenolic compounds.

**Table 3:** Effect of solvent type on the percent change of the recovery of phenolics, flavonoids, and antioxidants of black tea waste extracts

Solvent	TPC change (%)
Pure water	-
Mg water	59.70^c^
Hydrogen-rich water	193.05^a^
Ethanol/water (50/50)	-
Ethanol/Mg water (50/50)	77.56^b^
TFC change (%)	
Pure water	-
Mg water	33.46 ^c^
Hydrogen-rich water	210.56^a^
Ethanol/water (50/50)	-
Ethanol/Mg water (50/50)	82.41 ^b^
DPPH change (%)	
Pure water	-
Mg water	28.66 ^b^
Hydrogen-rich water	49.21^a^
Ethanol/water (50/50)	-
Ethanol/Mg water (50/50)	15.68 ^c^
ABTS change (%)	
Pure water	-
Mg water	58.25 ^b^
Hydrogen-rich water	86.60^a^
Ethanol/water (50/50)	-
Ethanol/Mg water (50/50)	12.29 ^c^

^a−d^ different lowercase letters show a significant difference between samples at *P* < 0.05

Molecular hydrogen dissolved in HRW possesses several specific physical, chemical, and physiological properties, such as antioxidant and redox homeostasis regulator (Alwazeer 2024a). Since the oxidation of phenolic substances can be restricted by reducing agents, such as hydrogen gas, the phenolic substances in HRW extracts were better protected and extracted by HRW than pure water. Similar results have been obtained by Ryu and co-workers (2019), who found that using hydrogen gas was more potent in recovering phenolic substances from fresh green tea leaves compared with other gases, including nitrogen, oxygen, carbon dioxide, and air (Ryu et al. 2019). The authors examined the morphological structure of tea leaves at the end of extraction, reporting that HRW-treated leaves have undergone a surface modification manifested by noticeable flexibility and smoothness characteristics that were not shown in other gases.

Many possible mechanisms were proposed to explain the improved impact of hydrogen-rich solvents on the recovery of phytochemicals, such as phenolic substances. These mechanisms include 1) the ‎protection of phenolics from oxidative reactions, 2) the reduction of quinones and semiquinones thanks to the reducing property of H_2_, 3) scavenging of the most reactive free radicals, i.e., hydroxyl radical‎ (^•^OH) and peroxynitrite (ONOO^−^), 4) scavenging of the polyphenol oxidase reaction products thanks to the selective antioxidant property of H_2_, ‎5) degassing dissolved oxygen, 6) ‎redox homeostasis of the plant cell cytoplasm, 7) modification of the ‎ physical structure of water, 8) activation of polysaccharide monooxygenases liberating the entrapped phenolic acids, ‎and 9) the modification of the surface structure of plant material (Alwazeer 2023). Hydroxyl radicals, formed via the Fenton reaction in the presence of ferrous, are strong oxidants that can almost immediately and unselectively oxidize all antioxidants and phenolics in the medium (Waterhouse and Laurie 2006). H_2_ has been proven to selectively scavenge hydroxyl radicals (Ohsawa et al. 2007).”.

Using Mg water that contains dissolved hydrogen, like HRW, led to an increase in the extraction of phenolic substances compared to pure water but less than HRW, with 59.70 and 193.05%, respectively (*P* < 0.05)(Table 3). This means that the presence of Mg^2+^ ions in the medium negatively influenced phenolic extraction, possibly due to the phenolic-Mg complex precipitation (Alwazeer et al. 2023d). Other possible mechanisms describing the negative impact of Mg on the recovery of phenolic substances from olive leaves were described in detail in a previous study (Alwazeer et al. 2023d).

### Total flavonoid content (TFC)

The TFC values of tea waste extracts prepared using different solvents are shown in Table 2. The solvent type significantly affected the TFC value (*P* < 0.05) (Table 3). Similar to the total phenolic content values, the best TFC levels were shown for HRW extracts (59.13 mg QE/g extract), followed by Mg water ones (25.41 mg QE/g extract)(*P* < 0.05). Additionally, using aqueous ethanol instead of pure water as a solvent effectively improved the recovery of flavonoids. Moreover, TFC values of ethanol/Mg water (50/50) (40.66 mg QE/g extract) were better than those of ethanol/water (50/50) (22.29 mg QE/g extract)(Fig. 1; Table 2).

Results showed that using HRW prepared by the infusion of H_2_ into water was the best method for extracting flavonoids from tea wastes, which increased by 210.56% compared with pure water extracts (*P* < 0.05). The method of Mg-water reaction for preparing HRW was also effective in extracting flavonoids but was less effective than the first method because it increased TFC by only 33.46%. However, the use of Mg water, which contains dissolved H_2_, instead of pure water in preparing aqueous ethanolic solvent (50/50, %vol) improved the extraction of flavonoids by 82.41% (*P* < 0.05) compared to ethanol/water (50/50, %vol) (Table 3). The above results show that the most effective method was using HRW prepared by H_2_ infusion for extracting flavonoids from tea wastes. The possible mechanisms behind this positive impact of H_2_ on the extraction of flavonoids can be explained similarly to those discussed above for phenolic substances since flavonoids form a subgroup of phenolic compounds.

### DPPH scavenging activity

The DPPH scavenging activity of tea waste extracts is shown in Table 2. Results show that the solvent type significantly affected the DPPH scavenging activity of tea extracts (*P* < 0.05). The tea waste extracts prepared using HRW showed the highest DPPH scavenging activity (22.65 mg AAE/g extract)(*P* < 0.05). Using aqueous ethanol instead of pure water as a solvent effectively increased the DPPH activity of tea waste extracts. Moreover, the use of HRW prepared by Mg-water reaction (Mg water) also showed higher DPPH scavenging activity than pure water (19.53 vs. 15.18 mg AAE/g extract, respectively) (Table 2).

Similar to phenolic and flavonoid results, HRW prepared by H_2_ infusion was more effective than that prepared by the Mg-water reaction method for extracting antioxidants from tea waste. Using HRW instead of pure water increased the DPPH scavenging activity of tea waste by 49.21% (*P*<0.0001), while the use of Mg water instead of pure water increased it by only 15.68% (*P*<0.0001) (Table 3). Additionally, using Mg water instead of pure water to prepare an aqueous ethanolic solvent positively improved the DPPH scavenging activity of tea waste extracts.

Again, these results of the DPPH scavenging activity of tea wastes can be explained by the positive impact of hydrogen on the stability of different antioxidants found in the plant cells thanks to the reducing properties of H2. The lower levels of DPPH scavenging activity of tea waste extracts in Mg water samples compared to HRW ones can be explained by the negative impact of Mg ions on the stability of many antioxidants like phenolics (Alwazeer et al. 2023d).

### ABTS scavenging activity

The ABTS scavenging activity of tea waste extracts prepared using different solvents is shown in Table 2. Results show that the tea waste extracts prepared by HRW had the highest ABTS scavenging activity (26.56 mg TE/g extract), followed by Mg water (22.55 mg TE/g extract) (*P* < 0.05). Additionally, using aqueous ethanol instead of pure water as a solvent effectively improved the ABTS scavenging activity of tea waste extracts. Moreover, the use of Mg water instead of pure water in the preparation of aqueous ethanolic solvent highly improved the ABTS scavenging activity of samples (Table 2). While Mg water increased the ABTS scavenging activity of tea waste samples by 58.25%, this value was increased by 86.60% when HRW was used instead of pure water. However, replacing pure water with Mg water for preparing an aqueous ethanolic solvent increased the ABTS scavenging activity by only 12.29% (*P* < 0.05). Results show that using HRW prepared by H_2_ infusion in pure water was better than HRW prepared by Mg-water reaction for extracting antioxidants. This may be due to the negative impact of Mg ions on the stability of some antioxidants, such as phenolic substances, as explained above.

### HPLC analysis

**Fig. 2:**
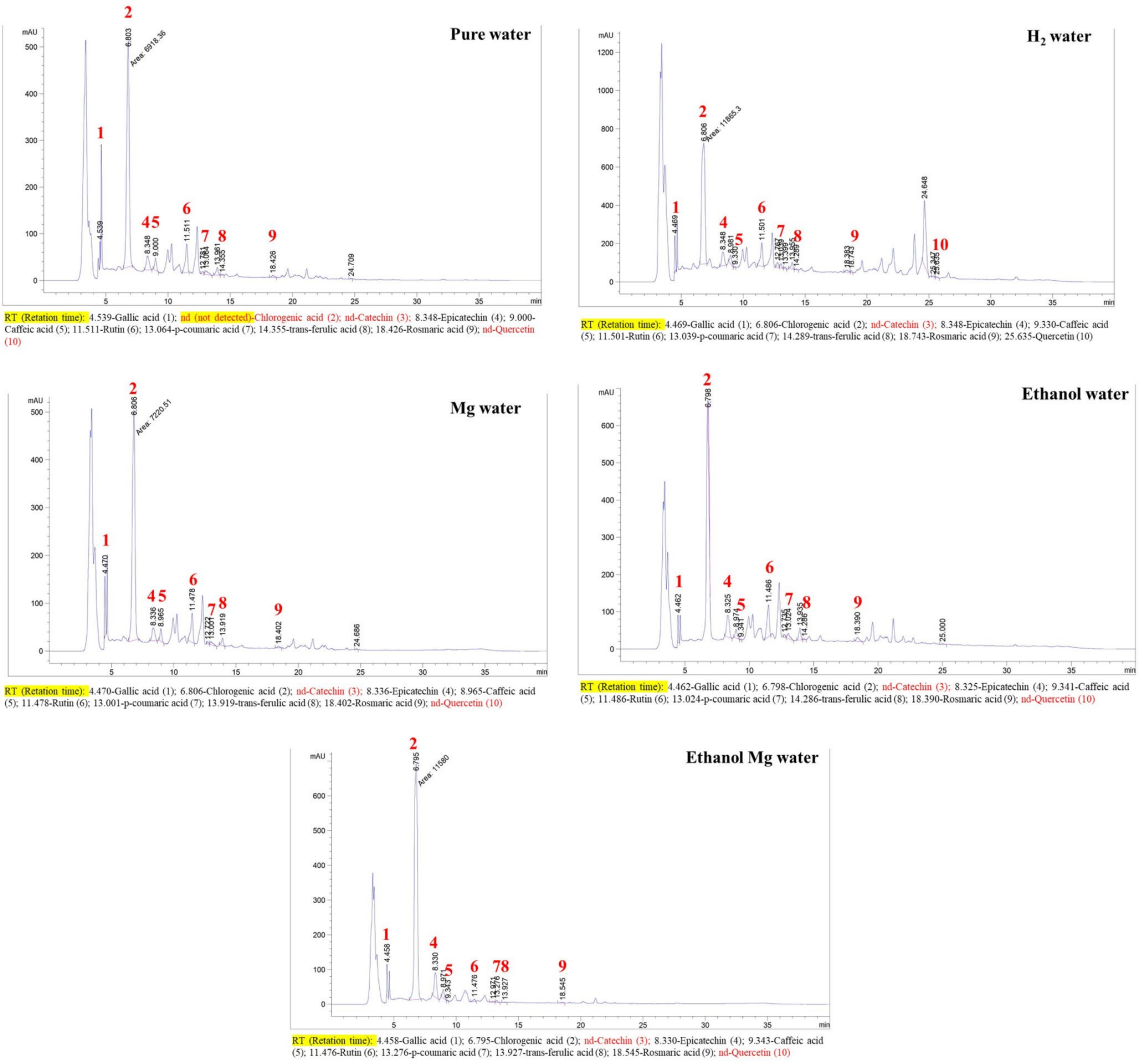
HPLC chromatogram of phenolic substances of tea waste samples

The phenolic profile of tea waste is shown in Table 4. Results show that some phenolic compounds, such as trans-ferulic acid, p-coumaric acid, and rutin, increased when HRW was used as a solvent in the extraction method (Fig. 2).

**Table 4:** Phenolic compounds of black tea waste extracts prepared by different solvents

Solvent	Phenolic Compounds (µg/g)									
	Gallic acid	Chlorogenic acid	*P*-coumaric acid	Trans- ferulic acid	Rosmaric acid	Caffeic acid	Catechin	epicatechin	Rutin	Quercetin
Pure water	15.787	nd	3.319	nd	4.207	6.345	nd	270.86	56.515	nd
Mg water	72.733	550.475	2.516	4.640	3.077	5.806	nd	247.986	54.417	nd
Hydrogen-rich water	109.407	904.325	4.687	1.626	4.836	11.211	11.232	612.418	108.87	nd
Ethanol/water (50/50)	37.554	81.673	4.262	nd	10.099	9.31	nd	492.579	66.43	1.749
Ethanol/Mg water (50/50)	46.086	882.59	1.562	nd	2.33	1.813	nd	561.327	77.785	1.980

On the other hand, other phenolic substances, such as catechin, appeared only in HRW extracts. Moreover, while chlorogenic acid was not found in the pure water extracts of tea waste, it appeared in HRW and Mg water samples with 904.32 and 550.47 µg/g extract, respectively. Additionally, when Mg water was used as a solvent, gallic acid levels increased by 360.71 µg/g, while when pure water was replaced with Mg water for preparing aqueous ethanolic solvent, the gallic acid increased by 22.72%.

## Conclusion

Tea waste forms a rich source of phenolic substances. The results showed that using hydrogen-rich water as a solvent can efficiently enhance the recovery of phytochemicals, including phenolic substances, flavonoids, and antioxidants from tea wastes. Using aqueous ethanol instead of pure water effectively improved the recovery of all phytochemicals from tea waste samples. Additionally, although using aqueous ethanol (50/50) could improve the phytochemical extraction, using Mg water instead of pure water for preparing the aqueous ethanol was more effective. The extraction yield was also improved by about two folds when HRW was used. HPLC analysis confirmed the spectrophotometric results, showing a significant increase in the levels of phenolics in HRW extracts, and some phenolics appeared only in this solvent’s extracts. The use of HRW to extract phytochemicals from agri-food wastes such as tea waste can be suggested as an eco-friendly and sustainable method. One of the limitations of the hydrogen extraction method is the potential explosion risk associated with hydrogen gas when it comes into contact with air (oxygen) at certain ratios. This risk can be managed with appropriate safety measures. Another challenge of this novel method is its non-selectivity towards various biochemicals, which necessitates post-treatment of the extract to remove undesirable substances. Further studies are required to address these challenges and to develop specific methods that target both the raw materials and the desired substances.

## Data Availability

The datasets during and/or analyzed during the current study are available from the corresponding author upon reasonable request.
